# *OsLBD3-7* Overexpression Induced Adaxially Rolled Leaves in Rice

**DOI:** 10.1371/journal.pone.0156413

**Published:** 2016-06-03

**Authors:** Cong Li, Xiaohua Zou, Chunyu Zhang, Qinghao Shao, Jun Liu, Bin Liu, Hongyu Li, Tao Zhao

**Affiliations:** 1 Institute of Crop Science, Chinese Academy of Agricultural Sciences, Beijing, 100081, China; 2 Shanghai Key Laboratory of Protected Horticultural Technology, Forestry and Fruit Tree Research Institute, Shanghai Academy of Agricultural Sciences (SAAS), Shanghai, 201403, China; 3 Jinan Licheng No.2 High School, Jinan, Shandong province, 250104, China; Universidad Miguel Hernández de Elche, SPAIN

## Abstract

Appropriate leaf rolling enhances erect-leaf habits and photosynthetic efficiency, which consequently improves grain yield. Here, we reported the novel *lateral organ boundaries domain* (*LBD*) gene *OsLBD3-7*, which is involved in the regulation of leaf rolling. *OsLBD3-7* works as a transcription activator and its protein is located on the plasma membrane and in the nucleus. Overexpression of *OsLBD3-7* leads to narrow and adaxially rolled leaves. Microscopy of flag leaf cross-sections indicated that overexpression of *OsLBD3-7* led to a decrease in both bulliform cell size and number. Transcriptional analysis showed that key genes that had been reported to be negative regulators of bulliform cell development were up-regulated in transgenic plants. These results indicated that *OsLBD3-7* might acts as an upstream regulatory gene of bulliform cell development to regulate leaf rolling, which will give more insights on the leaf rolling regulation mechanism.

## Introduction

Rice leaf shape or three-dimensional architecture plays important roles in the leaf’s function, including photosynthesis, respiration and transpiration [1, 2]. Moderate leaf rolling can improve photosynthetic efficiency, reduce sunlight damage to leaves and enhance leaf drought stress resistance, therefore strongly increasing yield performance. In contrast, serious leaf rolling could hinder plant development and severely reduce crop yield.

Most rolled leaves are related to leaf adaxial-abaxial polarity establishment and bulliform cell development [2–10]. Bulliform cells are groups of large and highly vacuolated cells that mainly exist in monocots and play a crucial role in the modulation of leaf rolling. It has been shown that the shrinking or expanding of bulliform cells contribute to the leaf rolling up or opening under different water stress conditions [11]. To date, several genes have been identified as leaf rolling genes in rice, which are associated with bulliform cell development. *Rice outermost cell-specific gene 5* (*OsRoc5*) is homologous to the *Arabidopsis* homeodomain leucine zipper class IV gene *GLABRA2* [3]. Suppression of *OsRoc5* causes abaxially rolled leaves and a reduced number and size of bulliform cells, whereas overexpression of *Roc5* results in adaxially rolled leaves and an increased number and volume of bulliform cells [3]. *SEMI-ROLLED LEAF1* (*OsSRL1)* encodes vacuolar H^+^-ATPase subunits and a H^+^-pyrophosphatase that regulates leaf rolling by repressing the development of bulliform cells [9]. Mutant *srl1* shows adaxially rolled leaves and an increased number of bulliform cells [9]. Rice *NARROW AND ROLLED LEAF1* (*OsNRL1*) encodes the cellulose synthase-like protein D4 (OsCslD4) [8]. *nrl1* mutant in rice shows semi-rolled leaves by reducing the size of bulliform cells [8]. *ADAXIALIZED LEAF1* (*OsADL1*) encodes a plant-specific calpain-like cysteine proteinase. *adl1* mutant plant results in abaxially rolled leaves due to the ectopic growth of bulliform cells [12, 13]. *ABAXIALLY CURLED LEAF1* (*OsACL1*) encodes an unknown protein of 116 amino acids [5]. Overexpression of *OsACL1* produces abaxial leaf curling and increases the number and size of bulliform cells [5]. *ROLLING-LEAF 14* (*OsRL14*) encodes a 2OG-Fe (II) oxygenase [7]. Mutant *rl14* exhibits incurved leaves and the shrinkage of bulliform cells on the adaxial side [7]. *SHALLOT-LIKE1* (*OsSLL1*) */ ROLLED LEAF 9* (*OsRL9*) encodes a *SHAQKYF* class *MYB* family transcription factor [2, 6]. *sll1* mutant displays an adaxialized leaves phenotype, while *OsSLL1* overexpression shows abaxial leaf features and suppresses bulliform cell development.

*LBD* gene family is a plant-specific transcription factor family, with 43 and 35 members in the *Arabidopsis* and rice genomes, respectively [14, 15]. *LBD* genes are involved in embryonic shoot apical meristem (SAM) formation and have an important influence on the formation and development of plant lateral organs, such as leaves, flowers and roots [14, 16, 17]. LBD proteins contain a characteristic LOB domain composed of a C-motif that is required for DNA-binding, a conserved glycine residue, and a leucine-zipper-like sequence that is required for protein-protein interactions [16]. The first identified *LBD* gene, *AtLOB*, was isolated from *Arabidopsis* in 2002 [14]. Ectopic expression of *AtLOB* exhibits smaller plant types and sterility. *AtAS2* (*AtLBD6*) is the second identified *LBD* gene in *Arabidopsis*. *AtAS2* is involved in symmetric flat leaf formation by inhibiting cell proliferation in the adaxial layer [18–20]. *AtAS2* is homologous to *OsAS2* (*OsLBD1-9*) in rice; *OsAS2* overexpression could inhibit shoot differentiation, promote cell division, delay cell differentiation and lead to aberrant twisted leaves [21].

In this study, we identified a novel rice rolled leaf gene that encodes a *LBD* family transcription factor. *OsLBD3-7* overexpression promoted narrow and adaxially rolled leaves by decreasing the size and number of bulliform cells. *OsLBD3-7* could also up-regulate bulliform cell negative regulators, which implies that *OsLBD3-7* acts as a suppressor of bulliform cell development.

## Methods

### Plant Materials and Growth Conditions

The rice cultivar Kita-ake (*Oryza sativa japonica* cv. Kita-ake) was used as the wild type line for phenotypic observation, histological analysis and gene expression analysis. In 2014 and 2015, all of the materials were grown in a field of the Experiment Station of Chinese Academy of Agricultural Sciences in Beijing (39°54′N, 116°23′E) under natural conditions from May to October of each year.

### Generation of Transgenic Rice Plants

To generate the *OsLBD3-7V* and *OsLBD3-7* constructs, full length *OsLBD3-7* was amplified from Nipponbare cDNA libraries and inserted into the gateway entry vector pDONR-201 (Invitrogen). Then, *OsLBD3-7* was recombined into destination vector pBCV [22] and pCAMBIA1301-Bar-FLAG using the Gateway cloning system (Invitrogen). The primers are listed in Table A in S1 File. The constructs were introduced into *Agrobacterium tumefaciens* strain *EHA105* and then transformed into Kita-ake wild type plants[23].

### *OsLBD3-7* Bioinformatics Analysis

The *OsLBD3-7* gene locus identifier is *LOC_Os03g57670*.*1*. Protein sequences were taken from the *Arabidopsis* (https://www.arabidopsis.org/) and rice (http://rice.plantbiology.msu.edu/) databases. Protein alignment was completed with ClustalX 2.0. A phylogenetic tree was constructed with MEGA5.1 using the Neighbor-Joining (NJ) method. The bootstrap values for nodes in the phylogenetic tree were from 1000 replications. The handling gap option was pairwise deletion, and the numbers at the branching points indicated the bootstrap values. The accession numbers and protein sequences were shown in Table B and C in S1 File.

### Leaf Rolling Index (LRI) Measurement

The mature flag leaves were measured in three replicates and each replicate included 10 individuals for each material. Lw was the greatest flag leaf width. Ln was the natural distance of the flag leaf margins at the same site. Leaf rolling index (LRI) was calculated with the following formula: LRI = (Lw-Ln)/Lw [10].

### Histological Analysis

The first and the second fully expanded leaves of 21 day seedling were collected, fixed in a FAA solution (60% (v/v) ethanol, 5% (v/v) glacial acetic acid and 5% (v/v) formaldehyde) and vacuum pumped for 40 minutes. Then, the leaves were dehydrated with a series of ethanol solutions (70% (v/v) ethanol, 80% (v/v) ethanol, 85% (v/v) ethanol, 90% (v/v) ethanol, 95% (v/v) ethanol, and anhydrous ethanol) and destained with a series of xylene solutions (3:1 ethanol: xylene, 1:1 ethanol: xylene, 1:3 ethanol: xylene, and pure xylene). The leaf tissues were soaked in each ethanol and xylene solution for two hours. Finally, the leaves were embedded in paraffin. Tissue sections were cut with a Leica rotary microtome, fixed on a glass slide, and stained with 0.05% Toluidine Blue O (Sigma).

### Subcellular Localization

Full length *OsLBD3-7* was inserted into the PA7-YFP vector, which had been digested with *Bam*HI and *Sma*I using the In-fusion system (Clontech). OsLBD3-7-YFP was transiently expressed in *Arabidopsis* mesophyll cells [24]. The AtAHL-RFP fusion protein was used as a nuclear marker. The fluorescence signal was observed under a confocal microscope (Zeiss LSM700) after 16 h of transformation at room temperature in the dark.

### Transactivation Activity Assays in Yeast

The full length *OsLBD3-7* was inserted into the pGBKT7 vector, which had been digested with *EcoR*I, and then was transformed into the yeast strain AH109. The pGBKT7 empty vector and BD-VP64 vector were used as controls. Transformed yeast was dropped onto SD/-W (-Trp) and SD/-W-H-Ade (-Trp/-His/-Ade) plates and allowed to grow for 48 hours before taking photographs. β-galactosidase activity was measured according to the Yeast Protocols Handbook (Clontech) using chlorophenol red-β-D-galactopyranoside as a substrate (CPRG, Roche Biochemical).

### RNA Isolation and qRT-PCR Analysis

Plants were grown under continuous light till four leaves stage and total RNA was extracted using the Trizol reagent (Invitrogen). The first complementary DNA was synthesized from DNase-treated total RNA (2 μg) with oligo (dT) primers using *TransScript*^®^ II One-Step gDNA Removal and cDNA Synthesis SuperMix (TransGen Biotech). qRT-PCR was performed using SYBR Premix Ex Taq (Takara) on a Roche Light Cycler 480 instrument. Quantitative reverse transcription PCR (qRT-PCR) experiments were performed with three replicates using the *Actin1* gene (*LOC_Os05g36290*.*1*) as the internal standard to normalize the expression of *OsLBD3-7* and the other tested genes. The gene expression levels (GEL) were calculated using the formula: [GEL = 2^-ΔCt^, ΔCt = Ct (Genes)-Ct (Actin1); Ct (cycle threshold) represents the number of cycles required for the fluorescence signal to exceed background level.

### Immunoblots

Immunoblot analysis was performed using one-week-old seedlings as described previously [25].

## Results

### *OsLBD3-7* Overexpression Displayed Rolled Leaf Phenotype

**Fig 1:**
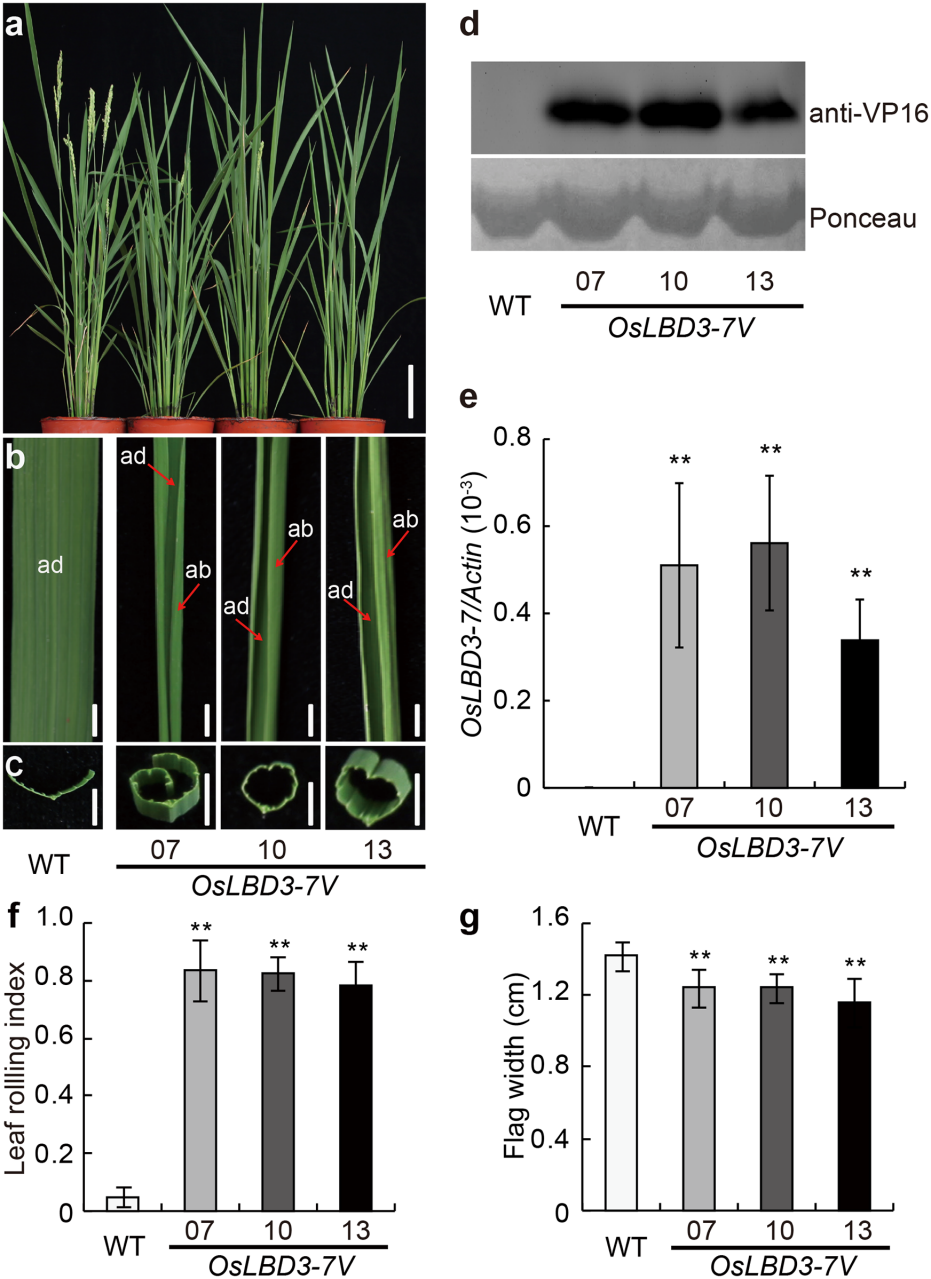
Phenotypic analysis of *OsLBD3-7V* overexpressing plants. **a** Gross morphology of WT and *OsLBD3-7V*. Bars = 10 cm. **b** The flag leaf morphology of WT and *OsLBD3-7V*. Bars = 5 mm. **c** Cross-sections of mature flag leaves. Bars = 5 mm. **d** Immunoblot analysis of wild type and *OsLBD3-7V* plants. **e** *OsLBD3-7V* expression level analysis by qRT-PCR. **f** LRI statistical analysis of flag leaves. **g** Flag leaf width statistical analysis. ab, abaxial; ad, adaxial. Data are shown as means ± s.d. (Student’s t tests, **P < 0.01, n = 3).

In our previous report [26], we utilized a hybrid transcription factor (HTF) approach that mimics the naturally occurring “exon shuffling” process [27] to fuse 1,500 rice transcription factors with the transcription activation module VP64 or repression module 4EAR, transforming them into rice accession Kita-ake and obtaining 50,000 transgenic rice lines. Among them, we identified an HTF, *OsLBD3-7V*, whose overexpression led to a rolled leaf phenotype. Three independent transgenic lines, *OsLBD3-7V-7*, *OsLBD3-7V-10* and *OsLBD3-7V-13*, which displayed different RNA and protein expression levels, were selected for further phenotypic analysis (Fig 1d and 1e). We first measured the LRI. The average LRI values for *OsLBD3-7V-7*, *OsLBD3-7V-10* and *OsLBD3-7V-13* flag leaves were 0.84, 0.83 and 0.78, respectively, while the wild type flag leaf LRI value was 0.05 (Fig 1f). Next, we measured the widths of the flag leaves. The results showed that *OsLBD3-7V-7*, *OsLBD3-7V-10* and *OsLBD3-7V-13* flag leaf widths were 1.24 cm, 1.15 cm and 1.25 cm, respectively, which were significantly narrower than the 1.42 cm wild type leaf (Fig 1g).

**Fig 2:**
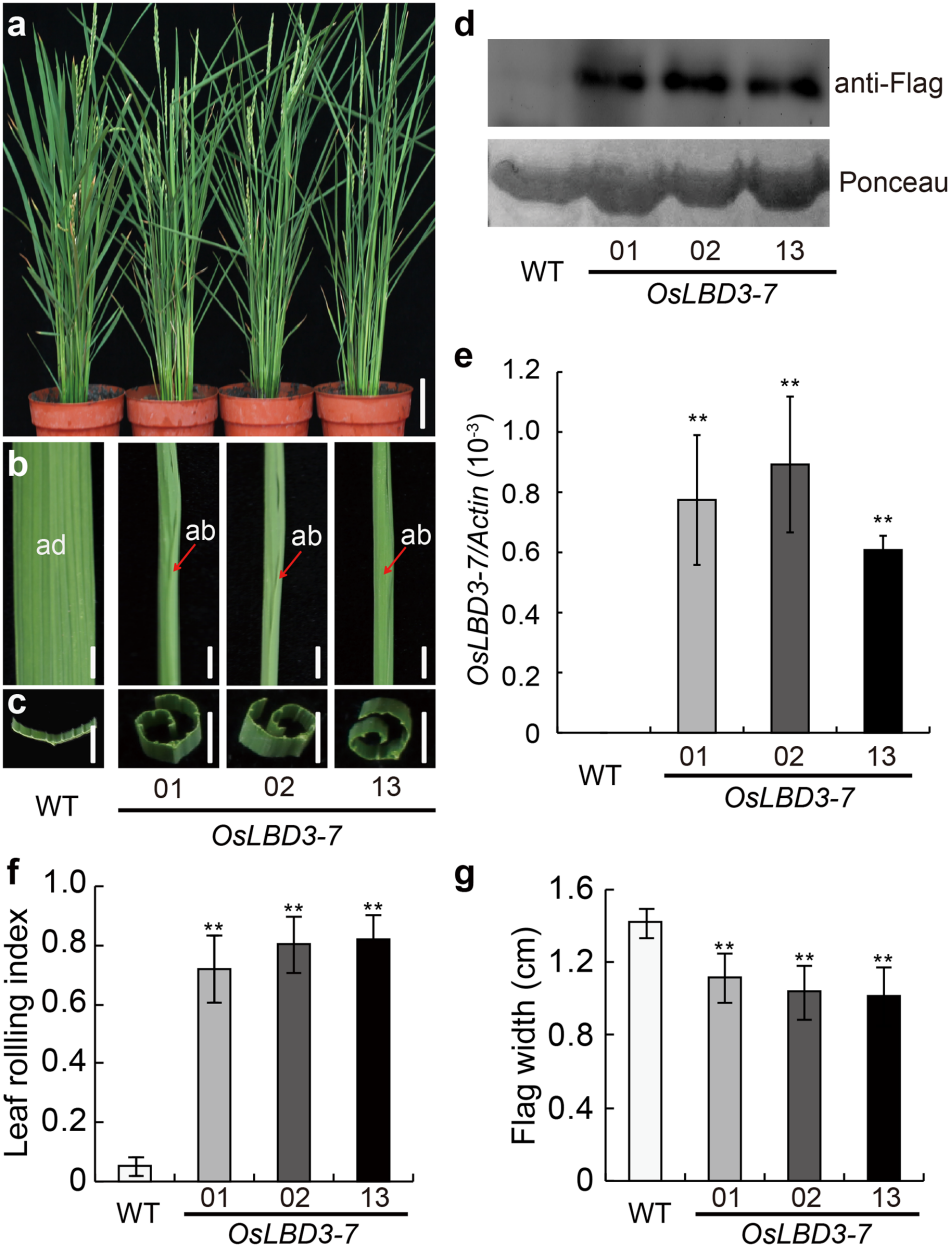
Phenotypic analysis of *OsLBD3-7* overexpressing plants. **a** Gross morphology of WT and *OsLBD3-7*. Bars = 10 cm. **b** The flag leaf morphology of WT and *OsLBD3-7*. Bars = 5 mm. **c** Cross-sections of mature flag leaves. Bars = 5 mm. **d** Immunoblot analysis of wild type and *OsLBD3-7* plants. **e** *OsLBD3-7* expression level analysis by qRT-PCR. **f** LRI statistical analysis of flag leaves. **g** Flag leaf width statistical analysis. ab, abaxial; ad, adaxial. Data are shown as means ± s.d. (Student’s t tests, **P < 0.01, n = 3).

However, the additional VP64 activation domain fused to OsLBD3-7 might lead to an artifactual phenotype. Thus, we constructed an *OsLBD3-7* overexpression vector under the control of the maize ubiquitin promoter (*Pubi*) and obtained 41 independent *OsLBD3-7* overexpression lines using an *Agrobacterium*-mediated transformation method. All of the transgenic lines also exhibited varying degrees of adaxially rolled leaf phenotypes (Fig 2). Taken together, these data show that *OsLBD3-7* overexpression induced narrow and adaxially rolled leaf phenotypes, while other phenotypic parameters remain unchanged compared with WT plants.

We also obtained *OsLBD3-7-RNAi* transgenic rice. None of these transgenic plants showed any obvious phenotypic changes during the entire rice growth period in comparison with wild type plants, although their mRNA levels were significantly reduced (data not shown).

### The Number and Size of Bulliform Cells in *OsLBD3-7V* Transgenic Plants Were Decreased

**Fig 3:**
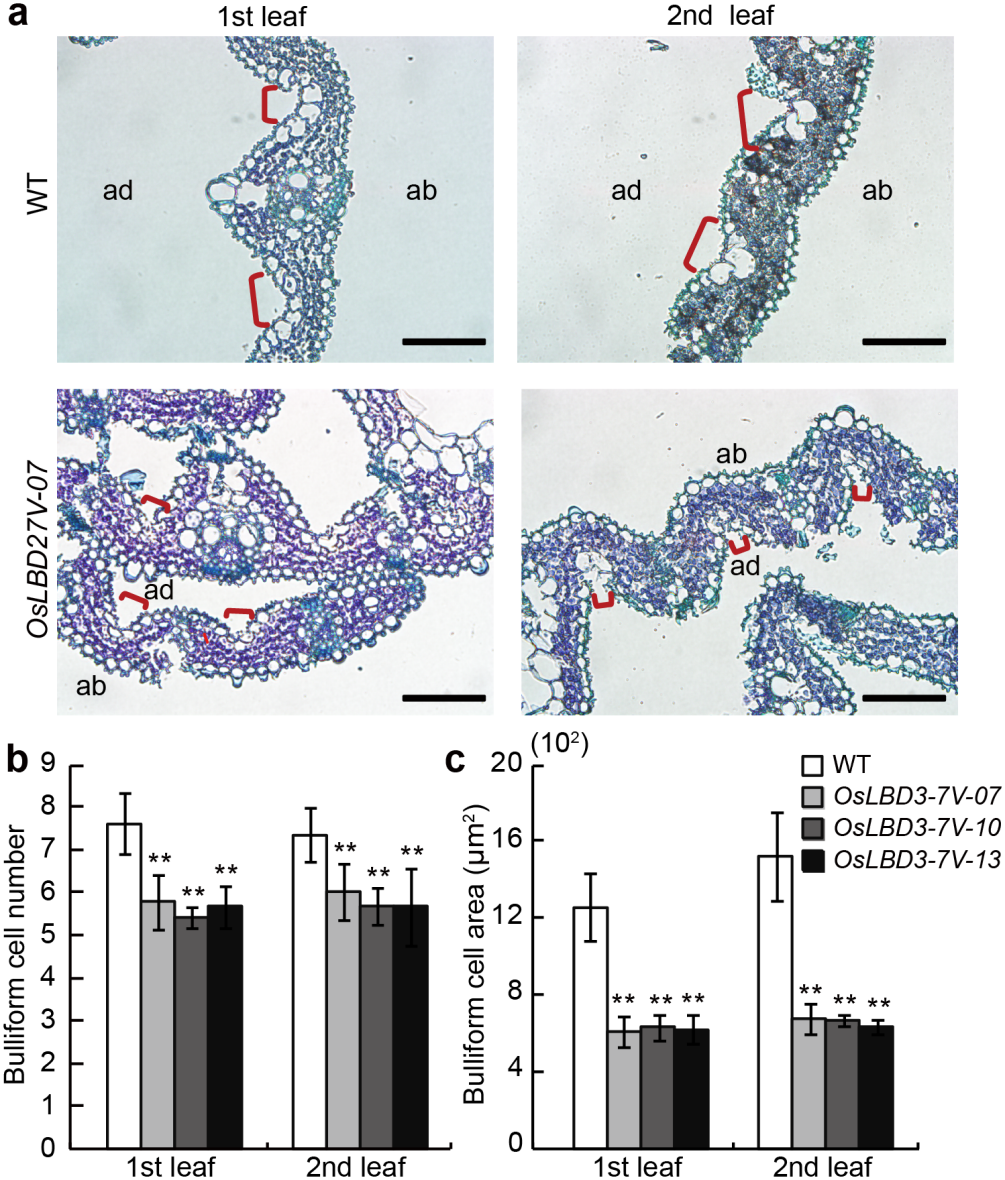
Histological observation of *OsLBD3-7V* seeding leaves. **a** Cross-sections of the first and second fully expanded leaves from 21-day-old wild type and *OsLBD3-7V* seedlings. ab, abaxial; ad, adaxial. Bars = 100 μm. **b** and **c** Bulliform cell number and area statistical analysis from wild type and *OsLBD3-7V* seeding leaves. Data are shown as means ± s.d. (Student’s t tests, **P < 0.01, n = 10).

To confirm that how overexpression of *OsLBD3-7* result in rolled leaf phenotype, we observed the detailed anatomy of wild type (WT) and *OsLBD3-7V* plant stretched leaves. The paraffin section results indicated that the *OsLBD3-7V* leaves’ abaxial epidermis exhibited no obvious differences compared with the WT. However, the number of bulliform cells in the adaxial epidermis of *OsLBD3-7V* leaves were remarkably reduced compared with WT leaves (Fig 3a). We counted the number of bulliform cells in the first and the second fully expanded leaves. Statistical analysis indicated that the number of *OsLBD3-7V* bulliform cells was 21% less than in the WT (Fig 3b). Furthermore, we measured the size of the bulliform cells in the *OsLBD3-7V* paraffin sections. The average values showed that the *OsLBD3-7V* bulliform cell size were 80% smaller compared with the WT (Fig 3c). These results indicated that the decreasing both bulliform cell size and number led to the rolled leaf phenotype.

### Rice *OsLBD3-7* Encodes a Typical LBD Family Transcription Factor

**Fig 4:**
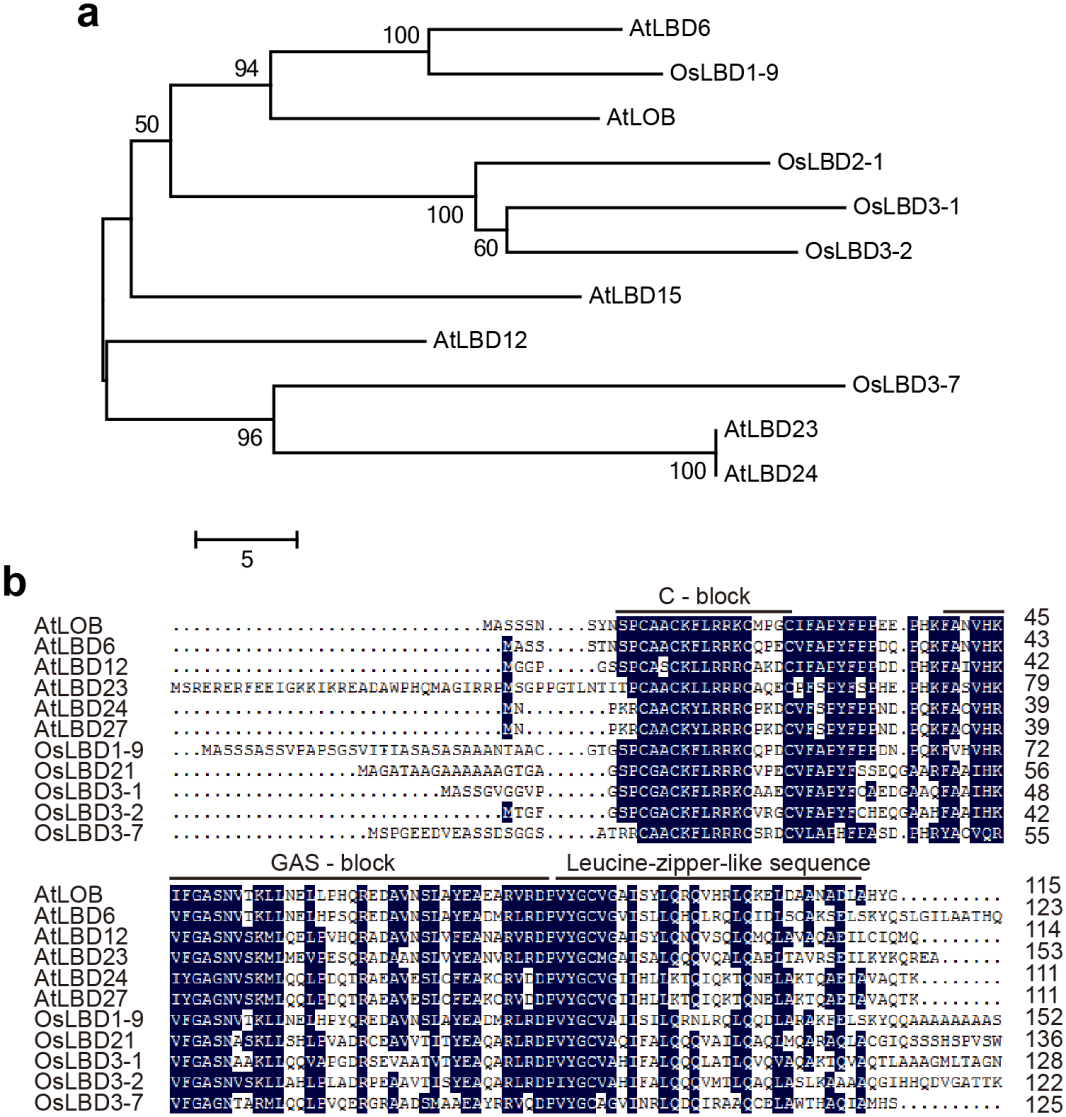
*OsLBD3-7* encodes a LBD family protein. **a** Phylogenetic tree analysis of the OsLBD3-7 protein with its rice and *Arabidopsis* homologues. **b** Amino acid alignment of the OsLBD3-7 protein and its *Arabidopsis* and rice homologues. Conserved amino acids are highlighted in blue.

Rice genome annotation analysis (http://rice.plantbiology.msu.edu/) showed that *OsLBD3-7* is located on chromosome 3 and encodes a 171 amino acid residues protein. Protein structural analysis revealed that OsLBD3-7 contains a C motif that has a 22 amino acid CX_2_CX_6_CX_3_C zinc finger-like domain, a GAS motif that starts with YX_2_VQ and ends with DP amino acids and a VX_6_IX_6_IX_6_L leucine zipper-like domain at the C-terminal (Fig 4b). Phylogenetic tree analysis showed that OsLBD3-7 shares high similarity with other homologues in rice (OsLBD1-9, OsLBD2-1, OsLBD3-1, and OsLBD3-2) and *Arabidopsis* (AtLOB, AtLBD6, AtLBD12, AtLBD23, AtLBD24 and AtLBD15) (Fig 4a). Amino acid alignment showed that *OsLBD3-7* shares 52% to 57% identity with *Arabidopsis* homologues, while sharing less than 45% identity with rice homologues (Fig 4b). Together, these data suggest that *OsLBD3-7* possesses the typical features of a Class I *LBD* family gene.

### Rice OsLBD3-7 Subcellular Localization and Expression Pattern

**Fig 5:**
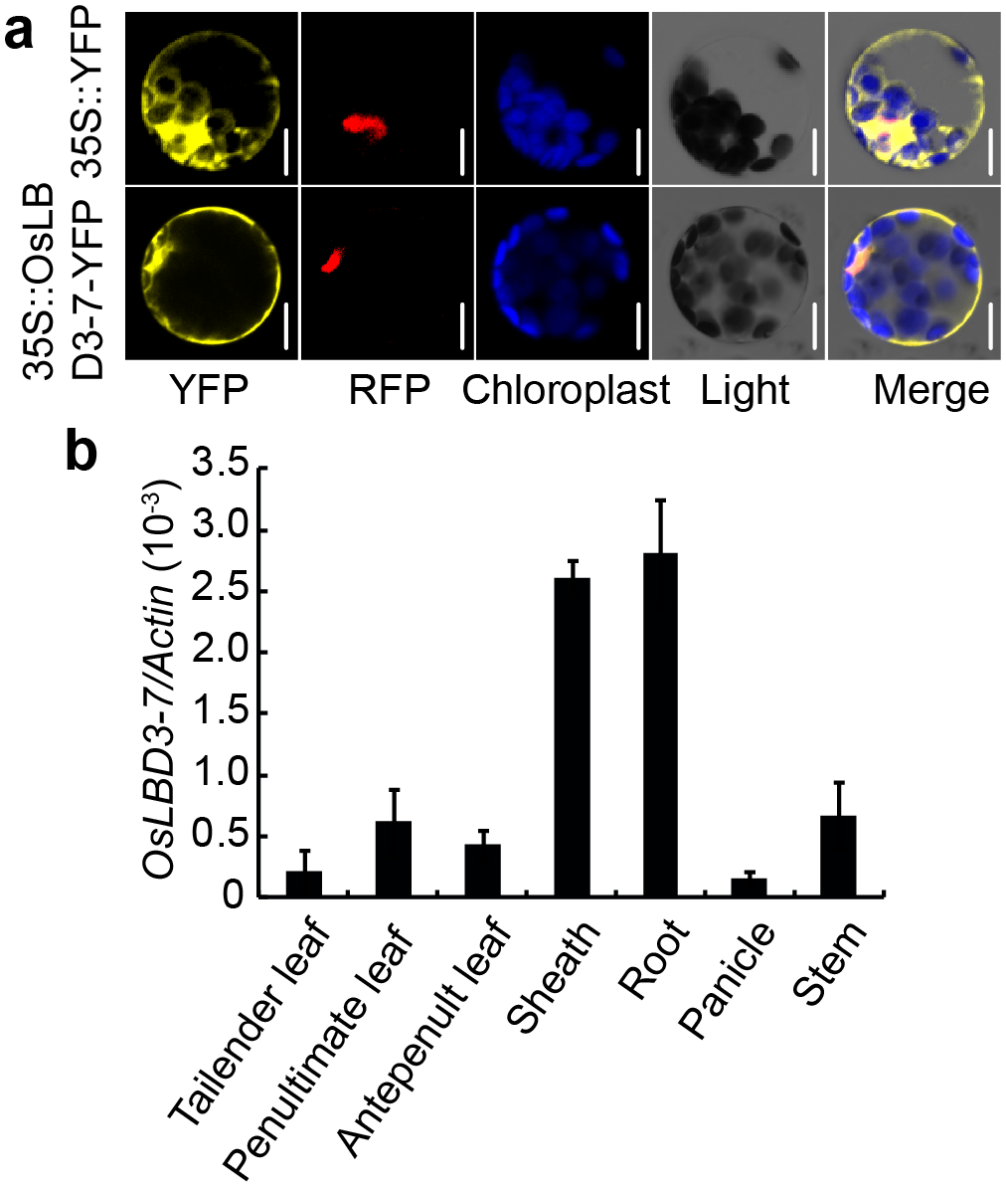
Subcellular localization and expression pattern analysis of OsLBD3-7 in rice. **a** Subcellular localization of OsLBD3-7. The OsLBD3-7-YFP fusion proteins were located on the plasma membrane and in the nucleus. YFP was used as a control. AHL-RFP fusion protein was used as a nuclear marker. Bars = 10 μm. **b** Expression pattern analysis of *OsLBD3-7* in various vegetative organs by quantitative RT-PCR. Data are shown as means ± s.d. (n = 3).

To examine OsLBD3-7 subcellular localization, we fused YFP to the OsLBD3-7 C-terminal. Transient expression in *Arabidopsis* mesophyll protoplasts showed that OsLBD3-7-YFP was exclusively located on the plasma membrane and in the nucleus. In contrast, the control protein YFP was only detectable in the intracellular region (Fig 5a).

To investigate the expression pattern of *OsLBD3-7*, we monitored its expression throughout its life period (from seeding to mature caryopses) with qRT-PCR. Our results showed that *OsLBD3-7* was ubiquitously expressed in almost all of the detected tissues, with especially high expression in the sheath and roots (Fig 5b).

### Rice *OsLBD3-7* Works as a Transcription Activator

**Fig 6:**
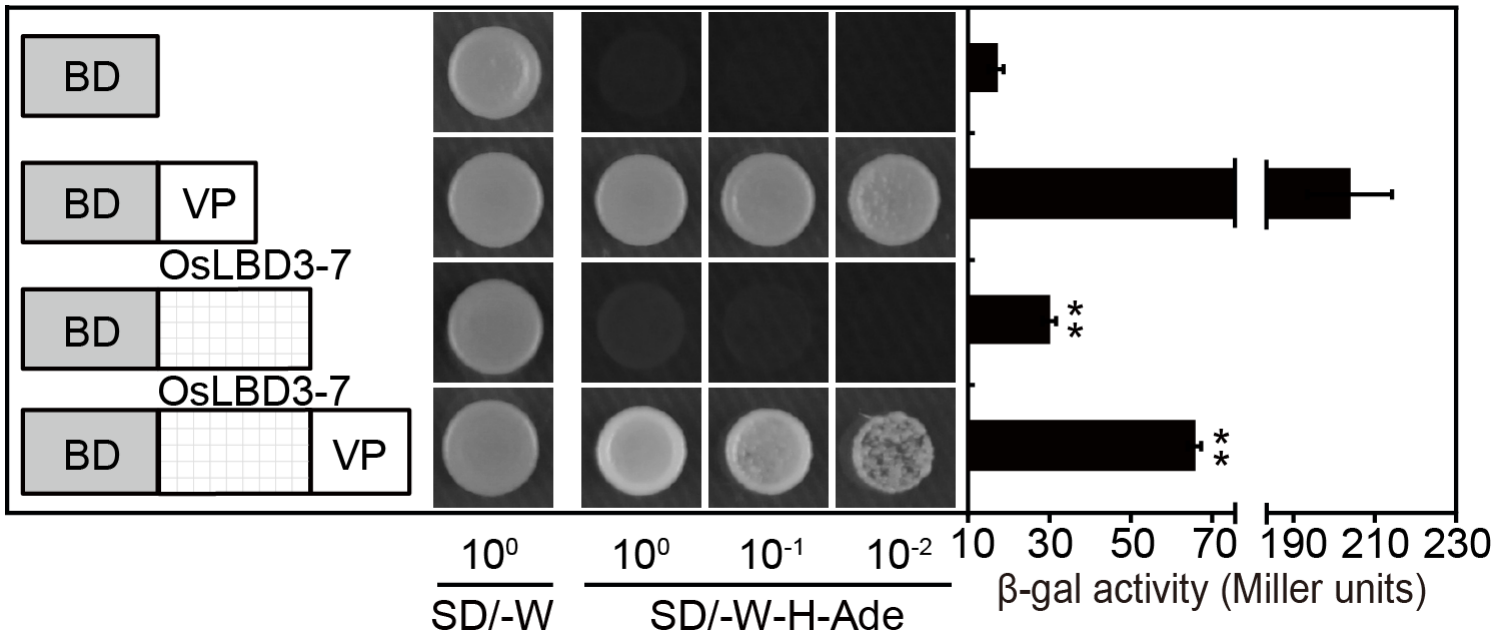
Transcription activation analysis of OsLBD3-7 in a yeast GAL4 system. Each transformed yeast strain was dropped onto SD/-W and SD/-W-H-Ade plates and allowed to grow for 48 hours before taking photographs. β-galactosidase activity was quantified through a liquid culture assay using CPRG as the substrate. Data are shown as means ± s.d. (Student’s t tests, **P < 0.01, n = 3).

Most characterized *LBD* genes have been reported to be transcription factors. To determine the transcriptional activity of OsLBD3-7, OsLBD3-7 and OsLBD3-7V were fused with a BD domain and transformed into the yeast cell AH109; the pGBKT7 empty vector and the BD-VP64 vector were used as controls. As shown in Fig 6, all of the yeast transformants grew well on the (-Trp) SD medium. When moved to (-Trp/-His/-Ade) SD medium, only BD-VP64 and OsLBD3-7V grew well, while OsLBD3-7 performance was a little better than the empty vector despite growth retardation. We also quantitatively monitored the β-galactosidase activity of all of the transformants using CPRG as substrate. The observed values showed that OsLBD3-7 possessed weak transcriptional activity, which was only two times higher than the empty control. However, when fused with the VP64 activation domain, OsLBD3-7 transcriptional activity increased dramatically (Fig 6). These results indicated that both OsLBD3-7 and OsLBD3-7V work as transcription activators, although OsLBD3-7V has a much higher activity than OsLBD3-7.

### Bulliform Cell Negative Regulation Genes Were Up-Regulated in the Rolled Leaf Plants

**Fig 7:**
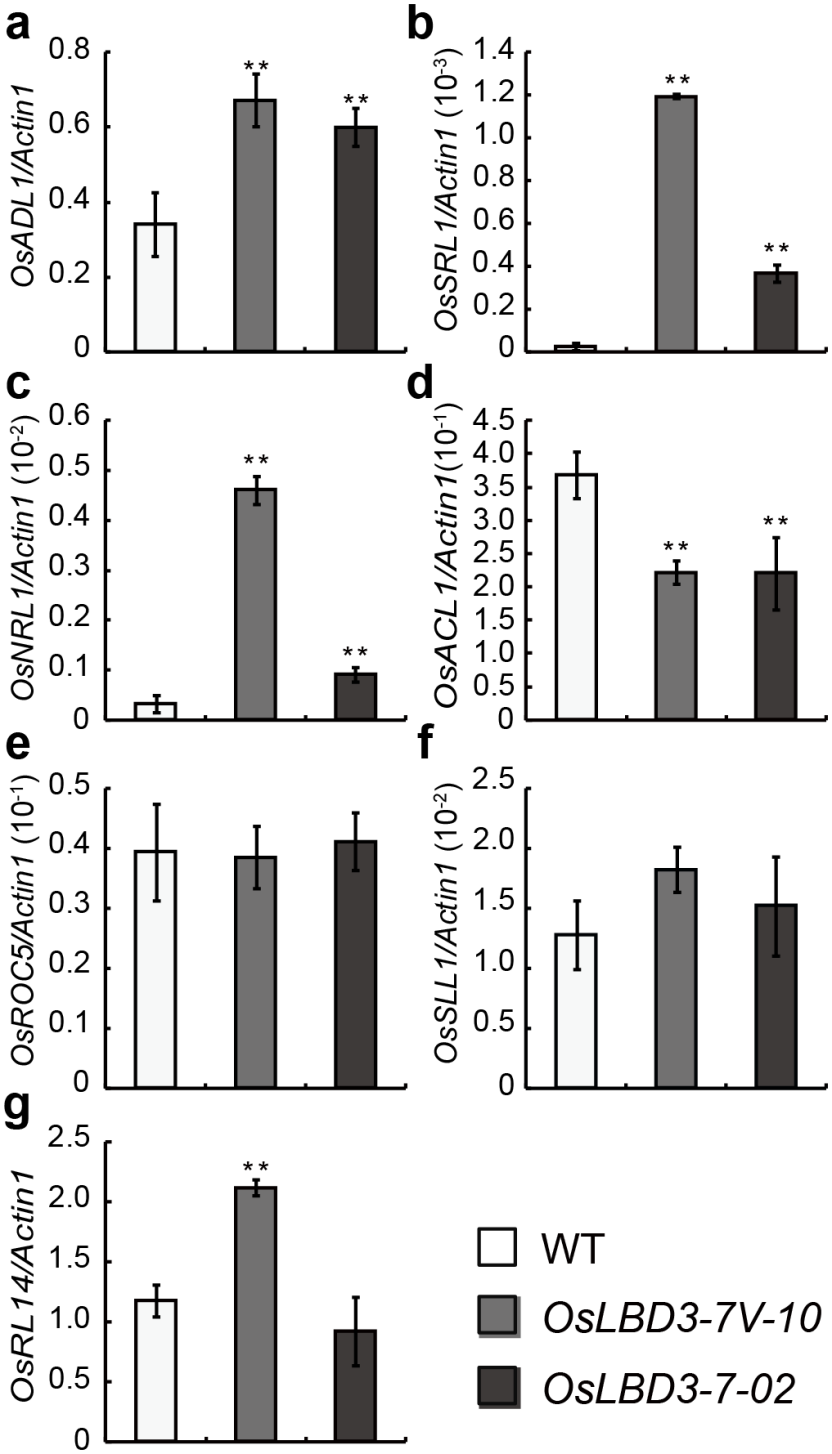
Expression analysis of bulliform cell regulation genes in *OsLBD3-7V* and *OsLBD3-7* overexpressing plants. **a** *OsADL1*, **b** *OsSRL1*, **c** *OsNRL1*, **d** *OsACL1*, **e** *OsROC5*, **f** *OsSLL1*, and **g** *OsRL14*. Data are shown as means ± s.d. (Student’s t tests, **P < 0.01, n = 3).

Our data indicated that the decrease in both bulliform cell size and number led to the rolled leaf phenotype, which suggests that *OsLBD3-7* is involved in bulliform cell development regulation. To confirm this speculation, we compared the mRNA expression of several key genes involved in bulliform cell regulation between the *OsLBD3-7*/*OsLBD3*-*7V* transformants and wild type seedlings at the four-leaf stage. The qRT-PCR results showed that the expression of *OsADL1*, *OsSRL1* and *OsNRL1* were significantly up-regulated (2–4 folds) in the *OsLBD3*-*7/OsLBD3*-*7V* transgenic seedlings compared with their respective expression in the WT, while *OsACL1* was significantly decreased (Fig 7). Therefore, the *LBD* family transcription factor *OsLBD3-7* might work as an upstream regulator of bulliform cell development to regulate leaf rolling.

## Discussion

Leaf size and shape are important morphological traits of plant architecture that affect the yield performance. Moderate leaf rolling could enhance erect-leaf habits, therefore improving the photosynthetic efficiency and increasing the grain yield. Although several genes have been reported to regulate leaf rolling, this complex trait is not yet fully understood. In this study, we reported a new rice *LBD* gene, *OsLBD3-7*, that might function in leaf development. *OsLBD3-7* is highly expressed in rice sheath and roots, and its protein is located on the plasma membrane and in the nucleus. *OsLBD3-7* overexpression could lead to adaxially rolled leaves, which are caused by the suppression of bulliform cell development.

Bulliform cells are a group of large parenchyma cells that specifically exist on the upper surfaces of leaves of many monocots. Bulliform cells are involved in young leaf unfolding, mature leaf rolling and unrolling in response to water stress conditions. Most rolling leaf mutants have been reported to be associated with the changes in bulliform cell number or size. Our data proved that *OsLBD3-7* overexpression could lead to cellular atrophy and a decrease in bulliform cell number. Previously, several leaf rolling/curling genes have been reported to be involved in bulliform cell development regulation. Our further investigations showed that *OsADL1*, *OsSRL1* and *OsNRL1* were up-regulated in *OsLBD3*-*7* and *OsLBD3*-*7V* transgenic plants, while *OsACL1* was significantly decreased. Among these genes, *OsADL1* and *OsSRL1* are bulliform cell negative regulators; mutants of these genes will lead to either bulliform cell number or size expansion. In contrast, *OsACL1* is a positive regulator, as *OsACL1* overexpression resulted in abaxial leaf curling and an increased number and volume of bulliform cells. Although *OsNRL1* is also a positive regulator, but only the mutants have semi-rolled leaves phenotype and over-expression plants have obvious phenotype on leaves. This might imply that *OsNRL1* and *OsLBD3-7* share different pathway. Together, the rolled leaf phenotype do not perfectly match with the expression of these leaf rolling/curling genes, but still suggests that the *LBD* family transcription factor *OsLBD3-7* might act as an upstream regulator of bulliform cell development to regulate leaf rolling.

The LOB domain comprises a C-domain that is presumably required for DNA-binding, which suggested that LBD genes act as transcription factors. Previous Research of *ASL4* in *Arabidopsis* has demonstrated that ASL4 is located in the nucleus and has the DNA-binding capacity to bind the GCGGCG motif [28]. Our data have indicated that *OsLBD3-7* is also located in the nucleus and has transcriptional activation activity. Thus, we further checked whether there was a GCGGCG motif in the promotor region (1,000 bps upstream) of *OsADL1*, *OsSRL1* and *OsACL1*. We identified at least one GCGGCG motif in the *OsSRL1* promotor regions, which suggests that *OsSRL1* might be the direct targets of *OsLBD3-7*.

## Supporting Information

S1 File Table A. Primers used in this study. Table B. Gene accession numbers in this study. Table C. The protein Sequences used to build phylogenetic tree.
(DOCX)
