# Supplementary material for: OsLBD3-7 Overexpression Induced Adaxially Rolled Leaves in Rice
Source: PLoS One. 2016 Jun 3;11(6):e0156413. doi: 10.1371/journal.pone.0156413 (PMC4892467; doi:10.1371/journal.pone.0156413)
Supplement: S1 File — Table A. Primers used in this study. Table B. Gene accession numbers in this study. Table C. The protein Sequences used to build phylogenetic tree. (DOCX) [file pone.0156413.s001.docx]

**Table A. Oligonucleotide primers used in this study.**

| **Experiment** | **Primer name** | **Primer sequence (5’-3’)** |
| --- | --- | --- |
| **Gene cloning** | attb-F | GTGGGGACAAGTTTGTACAAAAAAGCAGGCTTC |
|  | attb-R | GTGGGGACCACTTTGTACAAGAAAGCTGGGTC |
|  | attb-OsLBD3-7-F | CAAAAAAGCAGGCTTCATGTCCCCCGGTGAGGAG |
|  | attb-OsLBD3-7-R | CAAGAAAGCTGGGTCAGGAAACGAGAATTCAGATG |
|  | OsLBD3-7-PA7-F | CGAATTCCTGCAGCCCGGGATGTCCCCCGGTGAGGAG |
|  | OsLBD3-7-PA7-R | CACCATACTAGTGGATCCAGGAAACGAGAATTCAGATG |
|  | BD-OsLBD3-7 -F | CATGGAGGCCGAATTCATGTCCCCCGGTGAGGAG |
|  | BD-OsLBD3-7-R | GGATCCCCGGGAATTCAGGAAACGAGAATTCAGATG |
|  | BD-OsLBD3-7V -F | CATGGAGGCCGAATTCATGTCCCCCGGTGAGGAG |
|  | BD-OsLBD3-7V-R | GGATCCCCGGGAATTCTCAGAGCTCGGTACCGCT |
| **qRT-PCR** | OsLBD3-7-QF | GCCAGAATGCTCCAGCAAC |
|  | OsLBD3-7-QR | GTACACCGGGTCCTGCAC |
|  | OsSLL1-QF | GAACTTGGAACCGTGCAGAT |
|  | OsSLL1-QR | TCAGGCCTCCCTAGAGTGAA |
|  | OsROC5-QF | GTACCTTCCGGCTGTGTGAT |
|  | OsROC5-QR | CGAAGGAGTGGACGGTAGAG |
|  | OsADL1-QF | GGCGTGGTTATAGTGCAGGT |
|  | OsADL1-QR | AGTGCATCCCGTCCTGTTAC |
|  | OsSRL1-QF | GAGGACGACACCGAGATGAT |
|  | OsSRL1-QR | GAACCGCTTCGGTAGCATAG |
|  | OsNRL1-QF | GAGGGACTTCCTCAAGAACAAG |
|  | OsNRL1-QR | TCGTACTCGCGCTTCACCTT |
|  | OsACL1-QF | TCGCTGCTTCATCTACGTCA |
|  | OsACL1-QR | GTTGTTGTTGCCGTTGTCCT |
|  | OsRL14-QF | CGACTACAACGCCAAACTCA |
|  | OsRL14-QR | TTCTTGGAGGCAATGGATTC |
|  | OsACTIN1-QF | ATCCTTGTATGCTAGCGGTCGA |
|  | OsACTIN1-QR | ATCCAACCGGAGGATAGCATG |

**Table B. Gene accession numbers in this study.**

| **Gene Name** | **Gene ID** | **Mutant** | **Leaf Phenotype** | **Regulate Bulliform Cell** |
| --- | --- | --- | --- | --- |
| **OsADL1** | LOC_Os02g47970 | Loss-of-funcion | Abaxial leaf rolling | Negatively regulate |
| **OsSRL1** | LOC_Os07g01240 | Loss-of-funcion | Adaxial leaf rolling | Negatively regulate |
| **OsRoc5** | LOC_Os02g45250 | Loss-of-funcion | Abaxial leaf rolling | Negatively regulate |
| **OsSLL1** | LOC_Os09g23200 | Loss-of-funcion | Adaxial leaf rolling | Negatively regulate |
| **OsACL1** | LOC_Os04g33860 | Gain-of-function | Abaxial leaf rolling | Positively regulate |
| **OsNRL1** | LOC_Os12g36890 | Loss-of-funcion | Semi-rolled leaves | Positively regulate |
| **OsRL14** | LOC_Os10g40960 | Loss-of-funcion | Incurved leaves | Positively regulate |
| **OsZHD1** | LOC_Os09g29130 | gain-of-function | Abaxial leaf curling | Positively regulate |
| **OsSLL2** | LOC_Os07g38664 | Loss-of-funcion | Adaxially rolled leaf | Positively regulate |
| **OsCFL1** | LOC_Os02g31140 | gain-of-function | Curly flag Leaf | — |
| **OsCOW1** | LOC_Os03g06654 | Loss-of-funcion | Adaxially rolled leaf | — |
| **OsMYB103L** | LOC_Os08g05520 | gain-of-function | Adaxially rolled leaf | — |
| **OsAS2** | LOC_Os01g66590 | gain-of-function | Aberrant twisted leaf | — |
| **OsSRL2** | LOC_Os03g19520 | Loss-of-funcion | Incurved leaf | — |
| **OsAGO7** | LOC_Os03g33650 | gain-of-function | Adaxial leaf rolling | — |

**Table C. The protein Sequences used to build phylogenetic tree**

| **Gene** | **Protein Sequence** |
| --- | --- |
| **AtLOB** | MASSSNSYNSPCAACKFLRRKCMPGCIFAPYFPPEEPHKFANVHKIFGASNVTKLLNELLPHQREDAVNSLAYEAEARVRDPVYGCVGAISYLQRQVHRLQKELDAANADLAHYGLSTSAAGAPGNVVDLVFQPQPLPSQQLPPLNPVYRLSGASPVMNQMPRGTGGSYGTFLPWNNGHDQQGGNM |
| **AtLBD12** | MGGPGSSPCASCKLLRRRCAKDCIFAPYFPPDDPHKFAIVHKVFGASNVSKMLQELPVHQRADAVNSLVFEANARVRDPVYGCVGAISYLQNQVSQLQMQLAVAQAEILCIQMQNEPTLQSHHQVLELDQDHKALLLNNNNINNCNTNNNNNNFGYAMSSGQFNSNFASPSSIMQMQMQMQMQDPLKQESLWT |
| **AtLBD15** | MSRERERFEEIGKKIKREADAWPHQMAGIRRPMSGPPGTLNTITPCAACKLLRRRCAQECPFSPYFSPHEPHKFASVHKVFGASNVSKMLMEVPESQRADAANSLVYEANVRLRDPVYGCMGAISALQQQVQALQAELTAVRSEILKYKQREAVATLIVPSNSQVAGFHNSGGVSVIAPPPQRPTTPPQPTTAHPPSPSSCVFSQPTTRDLEYGNIESENNYFG |
| **AtLBD23** | MNPKRCAACKYLRRRCPKDCVFSPYFPPNDPQKFACVHRIYGAGNVSKMLQQLPDQTRAEAVESLCFEAKCRVDDPVYGCVGIIHLLKTQIQKTQNELAKTQAEIAVAQTKLSQTHISDFM |
| **AtLBD24** | MNPKRCAACKYLRRRCPKDCVFSPYFPPNDPQKFACVHRIYGAGNVSKMLQQLPDQTRAEAVESLCFEAKCRVDDPVYGCVGIIHLLKTQIQKTQNELAKTQAEIAVAQTKLSQTQNSDFM |
| **AtLBD6** | MASSSTNSPCAACKFLRRKCQPECVFAPYFPPDQPQKFANVHKVFGASNVTKLLNELHPSQREDAVNSLAYEADMRLRDPVYGCVGVISLLQHQLRQLQIDLSCAKSELSKYQSLGILAATHQSLGINLLAGAADGTATAVRDHYHHHQFFPREQMFGGLDVPAGNNYDGGILAIGQITQFQQPRAAAGDDGRRTVDPS |
| **OsLBD19** | MASSSASSVPAPSGSVITIASASASAAANTAACGTGSPCAACKFLRRKCQPDCVFAPYFPPDNPQKFVHVHRVFGASNVTKLLNELHPYQREDAVNSLAYEADMRLRDPVYGCVAIISILQRNLRQLQQDLARAKFELSKYQQAAAAAAAASASTGTNNGPHSMAEFIGNAVPNGAQSFINVGHSAALASVGGAAACFGQEQQFSAVHMLSRSYEGEPIARLGGNGGYEFGYSTSMAGGGHMSGLGALGGAPFLKSGIAGSDERQGAGQ |
| **OsLBD21** | MAGATAAGAAAAAAGTGAGSPCGACKFLRRRCVPECVFAPYFSSEQGAARFAAIHKVFGASNASKLLSHLPVADRCEAVVTITYEAQARLRDPVYGCVAQIFALQQQVAILQAQLMQARAQLACGIQSSSHSPVSWPDSGSISALLRQDMARRPPGGALDDCFGGGGALLPELMAAGFKDDVAAVQMQQHCSKAVDAGELQYLAQAMMRSTSNYSQ |
| **OsLBD31** | MASSGVGGVPGSPCGACKFLRRKCAAECVFAPYFCAEDGAAQFAAIHKVFGASNAAKLLQQVAPGDRSEVAATVTYEAQARLRDPVYGCVAHIFALQQQLATLQVQVAQAKTQVAQTLAAAGMLTAGNPLLQHQQQQQQAWQIEHESTMTSTQSSGCYSAPRSDGSTSLQDMYCFGEQEEGSYSR |
| **OsLBD32** | MTGFGSPCGACKFLRRKCVRGCVFAPYFCHEQGAAHFAAIHKVFGASNVSKLLAHLPLADRPEAAVTISYEAQARLRDPIYGCVAHIFALQQQVMTLQAQLASLKAAAAQGIHHQDVGATTKGGYMSAAATAADDQLGYGGYNQWCGSNGGGAPAASQPGAYSSNGGAGHGHDSITALLAAGSDYMQHSLYHAFEHSEGAGAVDDGHAAAAAFEAAAESSSCGMAASFAADESVWRSSSSGYQDCEDLQSVAYAYLNRS |
| **OsLBD37** | MSPGEEDVEASSDSGGSATRRCAACKFLRRRCSRDCVLAPHFPASDPHRYACVQRVFGAGNTARMLQQLPVQERGRAADSMAAEAYRRVQDPVYGCAGVINRLQDQIRAAQCELAWTHAQIAMHSAAAAHARTTLPPGQRDGGGGGAPSTQQATTSAAWQLEDFASEFSFP |
